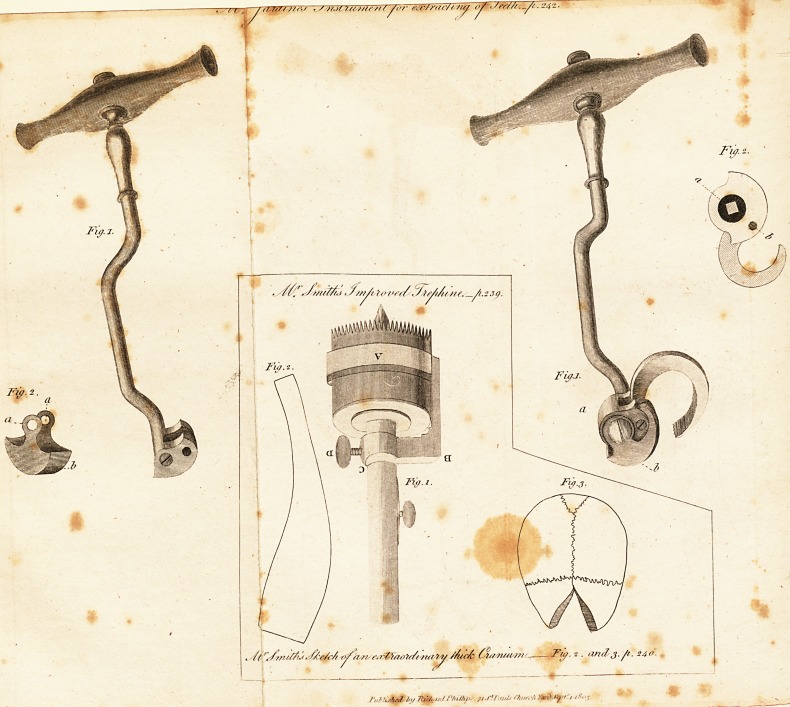# Mr. Jardine, on the Extraction of Teeth

**Published:** 1803-09-01

**Authors:** William Jardine

**Affiliations:** Dumfries


To Mr. BLAIR.

Sir,

THE interesting and impartial manner in which you seemed to examine the different instruments, which I lately proposed as improvements in the different operations for which which they were intended, persuade me, that the following description of the form of an instrument I have sent you, which I thought of for extracting of teeth, will not be unacceptable; and if you think it worthy of a place in the work you intend so soon to favour the world with, or in the Medical Journal of London, you will do me a favour in forwarding it to the Editors, or acquaint me by what means I may, myself, get it inserted in that periodical work.

I am rather doubtful you have not received all the satisfaction you may have expected, in reading the case of a collapsed brain of the man I trepanned on board the Victorious. I suspect it was very incorrect, and perhaps too prolix. I had not time, before I left London, to revise it; however, if you wish for a more correct and abridged copy, I will endeavour to send it you as soon as possible, and if you wish for a model of the tooth instrument I mentioned, I will likewise send it you by the first opportunity.

The descriptions of the instruments you saw, I find, have not yet been offered to public notice, in the Medical Journal of London, agreeably to Mr. Savigney's promise to me before I left town, and in a letter since to me at Dumfries. Indeed it was with much difficulty, since I came here, I could so much as procure an answer to the different letters I had occasion to write to him upon the subject.

The principal objection, I find, against the instruments alluded to, is the great expence they are likely to be to the purchasers. On that account, I mean to have them formed upon such a simple, and, as I think, on such an improved plan, as will not only reduce the price considerably, but make them more generally useful.

All the different instruments I have seen for extracting teeth, act upon such similar principles, and differ so little from one another, except in the shape or direction of the bolster, that it is difficult to judge which should have the preference.

It appears to me that the principal defect, in all the attempts to improve that instrument, has originated from the mistaken position of the claw on the top of the bolster, in preference to the position on the side. In that situation of the claw, (on the top of the bolster) it is very evident, that in turning the handle of the instument, for extractiug the tooth, 90°, the tooth must be lowered that much; whereas, if the claw is fixed on the side, supposing the bolster circular, or 90° from the top of the circumference, the shank being exactly in the centre of the bolster, the handle in being turned 90° in that operation, will raise the tooth as much, or the vertical height between the centre of the shank and pen of the claw: Wherefore, the nearer a right angle the claw, when fixed, makes with the top of the bolster, the higher the tooth will be raised.

Upon these principles I have directed an instrument to be made (*see plate*) with a bolster of a semicircular form, to receive the claw in its side, and to move in a slide, which will keep it up in its place when turned.

The slide is semicircular, except at the upper part that rests against the gum, when the instrument is fixed, which on the outside is flat, and it is attached to the bolster by a ring on each side, which the shank passes through. The bolster and slide are moveable upon the shank, that the instrument may be occasionally applied to either side of the mouth.

If the operator wishes to cover the face of the slide, in defence of the gum, there are two pins on each side of it, by which either cloth or leather may be tied to it.

I am afraid the enclosed draughts will not give you such satisfaction as I wish; they are very far from being what I expected, however, I have endeavoured, as well as I can with a pencil, to shew you the bolster detached, which is the principal part, and the most likely to give you the best idea of my intention.

I forgot to mention, in a former letter, that Mr. Rae a celebrated dentist in Edinburgh, made two successful trials of an instrument upon the same principle which I had sent him for that purpose, and that he meant to have one made for himself upon the same plan.

I might have acquainted you too, that I received a letter from Dr. Monro, since I arrived in Dumfries, acquainting me, that Mr. Fyfe, his assistant, had extracted a pin, which had stuck in the throat, with one of the same kind of instruments I had left with you for such a purpose.

*Dumfries,* I am, &c.

*January* 20, 1803. WILLIAM JARDINE.

## Figures and Tables

**Fig. 1. Fig. 2. Fig. 1. Fig. 2. f1:**