# Addenda

**Published:** 1803-09-01

**Authors:** 


### ADDENDA.

*Description of Mr. Jardine's Tooth Instrument*, (p. 241.)

PLATE I. Fig. 1, The instrument complete ; *a.* the bolster, *b.* the slide.
Fig. 2, The bolster detached ; *a.* the hole for receiving the shank, *b.* the pin-hole of the claw.

PLATE II. Fig. 1, A bad representation of the bolster and shank.

Fig. 2, The slide detached ; *aa.* the holes on each side of the slide through which the shank passes, *b.* the flat side of the slide that is placed against the gum.

